# Aging Brains Degrade Driving Safety Performances of the Healthy Elderly

**DOI:** 10.3389/fnagi.2021.783717

**Published:** 2022-01-25

**Authors:** Kaechang Park, Kazumi Renge, Yoshinori Nakagawa, Fumio Yamashita, Masahiro Tada, Yasuhiko Kumagai

**Affiliations:** ^1^Traffic Medicine Laboratory, Research Organization for Regional Alliance, Kochi University of Technology, Kami, Japan; ^2^Faculty of Psychology, Tezukayama University, Nara, Japan; ^3^Department of Management, Kochi University of Technology, Kami, Japan; ^4^Division of Ultrahigh Field MRI, Institute for Biomedical Sciences, Iwate Medical University, Morioka, Japan; ^5^Faculty of Science and Engineering, Kindai University, Higashiosaka, Japan

**Keywords:** driving safety performances, brain atrophy, leukoaraiosis, elderly drivers, dementia-free

## Abstract

The relationship between aging brains and driving safety performances (DSPs) of elderly drivers was studied. A total of 90 dementia-free participants (63 men and 27 women, mean age 75.31 ± 4.795 years) were recruited and their DSPs were analyzed on actual vehicles running through a closed-circuit course. DSPs were comprehensively evaluated on the basis of driving instructors' scores (DIS). Signaling and visual research behaviors, part of DSPs, were measured to supplement the DIS evaluation by driving recorders (DR) and wearable wireless sensors (WS), respectively. Aging brains were evaluated via magnetic resonance imaging (MRI) findings and experimentally assigned to two grades (high vs. low) of brain atrophy (BA) and leukoaraiosis (LA). Regression analyses on DIS and DR data, and logistic analysis on WS scores showed significant correlations of aging brains with degradation of DSPs. The participant group with more advanced BAs and LAs showed lower DIS, DR data, and WS scores representing degraded DSP regardless of age. These results suggest that MRI examinations from both volumetric and pathological perspectives of brains have the potential to help identify elderly drivers with dangerous driving behaviors. Brain healthcare, lifestyle improvements and medical treatments to suppress BA and LA, may contribute to preventing DSP degradation of elderly drivers with aging brains.

## Introduction

A recent surge in road traffic crashes caused by elderly (≥65 years of age) drivers has highlighted a call for action to better understand the underlying medical reasons and to mitigate the problem (National Police Agency, 2019). Better management of elderly drivers has become one of the top-priority issues in Japan where the population ratio of people ≥ 65 years of age is >28%: the highest in the world (Statistical Handbook of Japan, 2019). Therefore, great attention has been paid to the causal association between traffic crashes and phenomena associated with aging including declines in visual acuity (Yamamoto et al., 2020a), physical ability (Pavlidis et al., 2016), and executive functions (Taamneh et al., 2017). The influence of brain diseases, such as intracranial tumors (Mansur et al., 2018) or cerebral strokes (Aslaksen et al., 2013) on DSPs has also been investigated. DSP represents driving behavior that complies with traffic road regulations such as speed limits and signal stops at intersections through actual vehicle driving rather than use of a simulator. However, the brains of healthy aging drivers and their impacts on DSPs have only been studied as a social interest.

**Figure 1:**
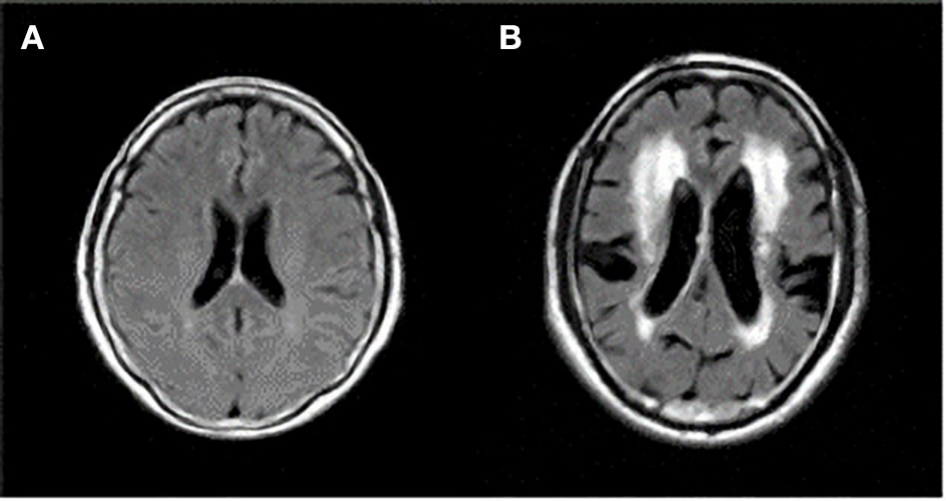
Axial magnetic resonance imaging (MRI) sections of two different brain features of healthy elderly individuals, both 70 years of age, who can drive normally. **(A)** Minimum of brain atrophy (BA) and leukoaraiosis (LA). **(B)** Larger BA with enlarged ventricles and sulci, and larger LA around ventricles. The corresponding author owns the copyright of these two MRIs.

Different changes appear in the aging brain. Aging is generally characterized by the shrinkage of certain areas of brain such as those important to learning and complex cognitive abilities (Seidler et al., 2010). Elderly people often display a deterioration in motor performance, and an important factor contributing to age-related performance decline is neurodegeneration which leads to psychomotor slowing and reduced fine motor control (Salthouse, 2000; Michely et al., 2018). Aging is also found in blood vessels resulting in vascular damages due to atherosclerosis (Schenk et al., 2012). These aging features in the brain generally manifest as BA and LA on MRI examinations (Guo et al., 2017), and large individual differences exist in the appearance of BA and LA among the elderly who can drive normally. Figure 1 shows typical examples of aging brains.

LA is regarded as ischemic lesions mainly produced by atherosclerotic changes of small blood vessels in the cerebral white matter. Recent functional MRI studies have reported functional difficulties due to LA. This is because the damaged vessels in LA induce an insufficient blood supply in the white matter including neuronal fibers, which can lead to dysfunction of neuronal network (Welker et al., 2012; Michely et al., 2018). LA has been reported to increase in frequency according to age and was found in at least 40–50% of apparently healthy adults over the age of 50, with some reports estimating as high as 95% (Grueter and Schulz, 2012; Schenk et al., 2012). However, most people have low grades of LA and exhibit no symptoms due to LA. In the case of intensive LAs, cognitive decline across many different kinds of functions is widely recognized (Schmidt et al., 2012; Macfarlane et al., 2013). Through the involvement of white matter communication channels, LA mainly affects executive functioning such as planning, prioritizing, risk assessment, processing speed, and attention that depend on the rapid and accurate exchange of information (Schmidt et al., 2012; Macfarlane et al., 2013), which can malfunction in elderly people (Miki and Sakamoto, 2013; Poggesi et al., 2013). These functions are involved in maintaining or improving DSPs (Aslaksen et al., 2013; Taamneh et al., 2017). In addition, our previous study with 3,930 healthy middle-aged drivers showed a significant association between the grades of LA and the incidence of traffic crashes in the past 10 years (Park et al., 2013). It was further reported that healthy elderly drivers with higher LAs produced more driving errors, such as ignoring stop signs, failing to confirm their safe right-of-way by checking left and right at intersections, and steering disturbances than do healthy LA-free elderly drivers under multitask loads: driving actual vehicles on a closed-circuit course while adding numbers that can be heard from their car stereo at 2-s intervals during driving (Nakano et al., 2014). These studies exhibited the clear impact of LA on DSP. However, during actual driving performances on a closed-circuit course without distracting tasks, LAs were not statistically correlated to a decrease in DSP when age was a confounding factor (Renge et al., 2020). The relationship between LAs and DSPs is still unclear.

On the other hand, BA has not been examined in terms of DSP degradation when using actual vehicles on the road. A voxel-based morphometry (VBM) study with 39 participants by Toyota Central Research using a driving questionnaire, not actual driving performances, revealed that reduced executive function capacity was significantly associated with reduced gray matter volume of the supplementary motor area (Sakai et al., 2012). Other cerebral sites were not investigated. Very recently, parts of DSPs were investigated using 32 participants and vehicle stability (acceleration and braking without knocking) at an intersection was reported to correlate to the volumes of several cortical regions including the superior frontal sulcus (Yamamoto et al., 2020b). Elucidating the relationship between brain volume and DSP has yet to be determined.

LA refers to white matter infarcts frequently leading to cerebral stroke and has multiple obvious causes. Hypertension is the strongest risk factor for LA (Thein et al., 2007; Choi et al., 2009). People who have hypertension are up to 14 times more likely to develop LA than those who do not have hypertension (Li et al., 2013). Obstructive sleep apnea (OPA) with intermittent breathlessness and snoring increases the risk of high blood pressure resulting in LA (Culebras, 2004). Obesity is a leading risk factor for OPA, and physical exercise and low-calorie diets are the most effective countermeasures. Other risk factors for LA include diabetes (Park et al., 2009; Putaala et al., 2009), metabolic syndrome (Park et al., 2007; Bokura et al., 2008), and smoking (Longstreth, 2005; Gons et al., 2011). Comprehensive countermeasures include blood pressure control with or without drug treatment, suppression of excessive salt intake, regular physical exercise, dietary control, and a cessation of smoking. On the other hand, advancing age appears to cause BA. In addition to aging, alcohol drinking and smoking are known to cause a reduction in brain volume (Paul et al., 2008; Elbejjani et al., 2019). In particular, alcohol intake shrinks brain volume even if the amount of alcohol consumption is not excessive (Paul et al., 2008). Moderate drinking and no smoking suppress brain volume shrinkage. In summary, improved lifestyle habits and drug treatments such as antihypertensive remedies can suppress BA and LA which can possibly retard brain aging.

**Figure 2:**
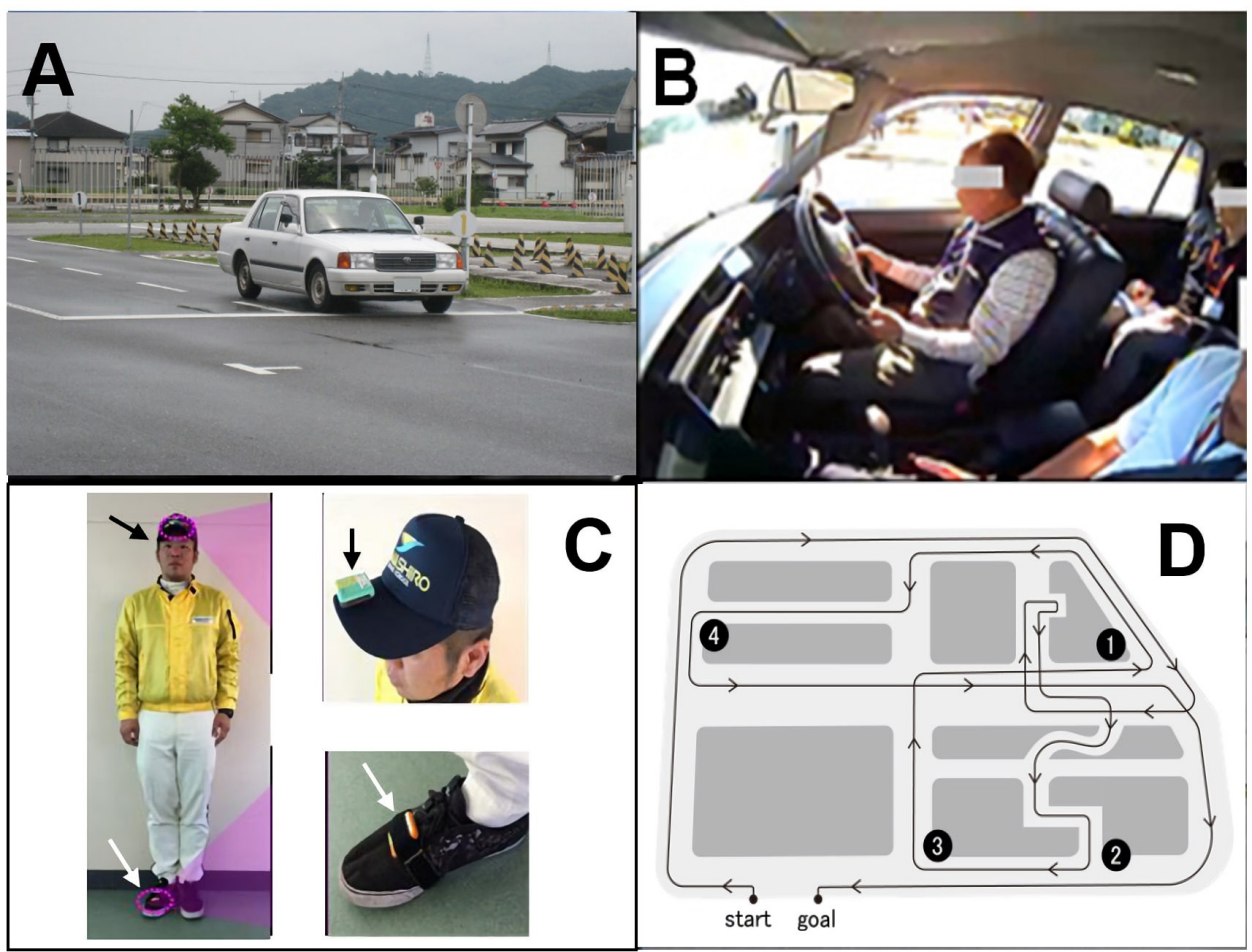
Images of the wearable wireless sensor (WS) and an actual vehicle on the driving experimental course. **(A)** vehicle overview, **(B)** in-car view with the driving recorder (DR), **(C)** WS on the cap (black arrow) and the right shoe (white arrow), **(D)** driving course map with four locations. ①: left turn, ②: right turn, ③: right turn without stop sign, ④: left turn with stop sign. The copyright of all photographs belongs to the corresponding author.

This evidence indicates that healthcare or medical countermeasures evaluated by MRIs may be effective in decreasing dangerous driving behaviors caused by elderly drivers. Therefore, the present study examined the individual and combined influences of BA and LA on DSPs when driving actual vehicles (Figure 2).

## Materials and Methords

### Participants

A total of 90 participants (63 men and 27 women, mean age 75.31 ± 4.795) were enrolled in the present study. They were recruited in Brain Dock, one of preventive medicine fields which has been uniquely prevalent in Japan as previously described (Nakano et al., 2014). Each participant received an MRI examination as a part of a healthcare checkup in Brain Dock at the Kochi Kenshin Clinic, which is affiliated with Kochi University of Technology (KUT). The average mini-mental state examinations (MMSE) scores were 28.19 ± 1.609 (24–30; median: 28). A dementia medical specialist diagnosed all participants as non-dementia. They had no cerebrovascular diseases or brain tumors. The driving experience and exposure of participants were conditioned for the enrollment as follows; all drove > 2 times per week and 5 km per week to work-sites, shops, and hospitals. Professional drivers were excluded in the present study.

The 90 participants were divided into two groups according to their aging brain levels with BA and LA evaluated by MRI. The higher aging brain group consisted of participants with BA medians > 0.2171, and LA grades above 2. The lower aging brain group consisted of the participants with BA medians < 0.2171 and/or LA grades of 2.

### Measurement

#### MRI Examination

A 1.5-Tesla MRI ECHELON Vega system (Hitachi Medical Corporation, Tokyo, Japan) was used for the detection of LA. The imaging protocol included T2-weighted spin-echo [repetition time/echo time (TR/TE) = 5,800/96 ms], T1-weighted spin-echo (TR/TE = 520/14 ms), and FLAIR (TR/TE = 8,500/96 ms; inversion time = 2,100 ms) images. LA was defined as a focal lesion or periventricular layer with a diameter ≥ 2 mm, hyperintensity on T2-weighted and FLAIR images, and without prominent hypointensity on T1-weighted images. The thicknesses of MRI slices were 5 mm, and three sections of the axial, sagittal, and coronal 2-D views were used for LA examinations.

#### Grading of Leukoaraiosis

Radiological evaluations of the participants' LA were made using three kinds of MRI images: T1-weigted, T2-weighted, and FLAIR images. They are useful for differential diagnoses of LA from cerebral infarction and brain tumors (Zhu et al., 2020). LA was classified into six grades from G0 to G5 according to previously delineated criteria (Putaala et al., 2009; Park et al., 2013). Briefly, G0 was defined as no lesions, G1 as one minimal dot (2–3 mm) of LA, G2 as two minimal dots, G3 as three or more scattered dots in the subcortical medulla area, G4 was defined as 3–5 mm periventricular layers or patches including G3, and G5 as periventricular layers or patches > 5 mm, such that the higher the grade, the more progressive the lesion.

#### Measurement of Brain Atrophy

T1-weighted MRI images were obtained using a 1.5 Tesla ECHELON Vega system (Hitachi, Tokyo, Japan) with three-dimensional gradient-echo with inversion recovery (3D-GEIR) sequences. The following scanning parameters were used: repetition time, 9.2 ms; echo time, 4.0 ms; inversion time, 1,000 ms; flip angle, 8°; the field of view, 240 mm; matrix size, 0.9375 × 0.9375 mm; slice thickness, 1.2 mm; and the number of excitations = 1. Each image was visually assessed for brain diseases and anomalies; head motion and artifacts are factors which affect the volumetric measurement. The images were processed and analyzed using the VBM8 toolbox (http://dbm.neuro.uni-jena.de/vbm/) and other modules implemented in the Statistical Parametric Mapping (SPM) 8 (https://www.fil.ion.ucl.ac.uk/spm/) to estimate regional cerebral volumes (Sasaki et al., 2008; Whitwell, 2009). Images were segmented into gray matter (GM), white matter (WM), and cerebrospinal fluid space. The segmented GM and WM images were then used to estimate the morphological correspondence between the template image and the participant's brain using a high-dimensional non-linear warping algorithm (Kurth et al., 2010). The estimated non-linear warp was inversely applied to an atlas defined in the template space to anatomically parcellate the target brain. The Neuromorphometrics atlas incorporated in SPM12 was used for the parcellation, with a modification for WM lesions, which appeared as incorrect GM segments around the lateral ventricles. Each anatomical region volume was calculated as the sum of the correspondent tissue densities in the voxels belonging to each region. Intracranial volume (ICV) amounts to the summation of total brain volume (TBV) and cerebrospinal fluid (CFV). BA was defined as the ratio of CFV to ICV ((ICV – TBV)/ICV = 1 – TBV/ICV).

#### Evaluation by Driving Instructors' Scores

Actual vehicle driving experiments were performed on a closed-circuit course, officially designated for renewing drivers' licenses for elderly drivers by the National Police Agency (The Driver's License Skill Test Implementation Standard Available at National Police Agency, 2016), in a driving school in Kochi City, Japan (Figure 2A) (Renge et al., 2020). The driving course has various locations where driving performance is evaluated, e.g., right and left turns, S-shaped curve, crunch, right and left curves, pauses according to stop signs at intersections, and lane changes. In the present test, four locations on the driving course were selected for rating. These locations included intersections with two right turns, one left turn having restricted visibility and no stop signs, and another left turn having a stop sign (Figure 2D). A Toyota-made four-wheeled 1400cc vehicle (COMFORT) was used. The typical speeds of the vehicles on the closed-circuit course are 30–50 km/h and it takes ~20 min to complete a circuit. Official driving instructors can accomplish the evaluation after 2 right turns and 2 left turns. No further driving events were embedded in the test. In advance of the test, a qualified driving instructor drove around the course, demonstrating good driving performance with a participant sitting in the seat next to the instructor. Then, the participant drove with the evaluating instructor sitting in the passenger's seat. The official instructors rated the driving skills of each participant using the previously described method (Renge et al., 2020). They responded to the items using a five-point scale: 1 (not done), 2 (poorly done), 3 (normally done), 4 (well done), and 5 (very well done). These rating scores at four locations were then calculated as the “overall evaluation” by assessing the six categories: “visual search behavior (safety recognition with head movement),” “speeding (choice of vehicle speed),” “signaling (indicator),” “vehicle stability (acceleration and braking without knocking),” “positioning (vehicle movement along the radius of curvature at intersections without large or small turns),” and “steering (smooth handling with appropriate starting and ending).” Larger scores indicate stronger compliance with the Road Traffic Act. An average value of the summed scores at the four locations for the two rounds of the course was calculated for the DIS.

#### Evaluation by Driving Recorders

After the driving instructors scored the DSPs, DR data were collected and analyzed. As previously described, the duration times of directional signaling with blinkers, from the entrance to the exit of three locations including one right turn and two left turns (①③④ in Figure 2D), were measured with DR images and timers (KDR-4CU by Kawasaki Kogyo Co., Ltd.) (Renge et al., 2020). The summation of duration times at the three locations was later conducted through the imaging playback with DRs. The instructors had told participants to operate blinkers 30 m before arriving at the three locations according to the Road Traffic Act. Longer times were regarded as safer signaling.

#### Evaluation by Wearable Wireless Sensors

Visual research behaviors at these four locations as well as the instructor's evaluations were assessed using WS (OBJE by ATR-Sensetech, Japan) composed of a three-axis gyro chip and a three-axis accelerometer chip (Renge et al., 2020). As described in Figure 2, a WS was attached to the driver's head with a special cap and two others were placed on the participants' right shoe and the central dashboard of the vehicle. Angles and durations of the drivers' head movements during the visual search at intersections were scored on a scale ranging from 0 to 10 points. WS scores were obtained by the sum of points at one location (④ in Figure 2) for the two trips around the course. WS allowed the evaluation of safe driving behaviors at potentially dangerous spots by measuring and analyzing head-motion data (precision ratio: 83.2%, recall ratio: 81.7%) (Tada et al., 2014). Larger scores were regarded as safer behaviors of drivers for visual research (Renge et al., 2020).

#### Statistical Analysis

Independent *t*-tests were used to evaluate the difference in mean values of participants' profiles between high- and low-grade coupling of BA and LA. Multivariate linear and logistic regression analyses were used to evaluate subjects' DSPs in terms of age, gender, and brain conditions characterized by the degrees of LA and brain volumes. Three driving performance indices were adopted: DIS (the scores evaluated by driving instructors for multivariate linear regression analysis), DR data (the duration times of signaling as measured by the driving recorder for multivariate linear regression analysis), and WS scores (angles and durations of the drivers' head movements measured by WS for logistic regression analysis). In analyzing WS scores, logistic regression was applied considering the frequency distribution of the scores. As shown in Figure 2C, a number of subjects had the maximal or minimal scores in the theoretical range.

## Results

### Comparison of Participants With Higher and Lower Grades of Aging Brains

In the present study, 35 participants had higher grades of aging brains (those who satisfy both LA ≥ 2 and BA ≥ 0.217 as median values), and 55 participants had lower grades of aging brains (Table 1). Mean age, LA grading, CFV, and BA were significant while mean MMSE scores, TBV, and ICV were not.

**Table 1:** The profile of participants with higher and lower grades of aging brains.

	**Higher (*n* = 35)**	**Lower (*n* = 55)**	**Higher vs. lower**
Participants' ages	78.43 ± 4.146	73.44 ± 4.167	^*^
MMSE (24–30)	27.49 ± 1.991	28.55 ± 1.412	ns
LA (0–5)	3.49 ± 0.818	2.11 ± 1.560	^*^
TBV (cm^3^)	1,033.69 ± 81.940	1,068.31 ± 105.275	ns
CFV (cm^3^)	316.48 ± 35.091	280.64 ± 91.940	^*^
ICV (cm^3^)	1,350 ± 108.244	1,350 ± 129.309	ns
BA = 1 – TBV/ICV	0.234 ± 0.014	0.209 ± 0.015	^*^

*MMSE, mini mental scale examination; LA, leukoaraiosis; TBV, total brain volume; CFV, cerebrospinal fluid volume; ICV, intracranial volume; BA, brain atrophy; ns, not significant; higher, both LA ≥ 2 and BA ≥ 0.217*.

* *p < 0.05*.

### Scatter Plots of Driving Instructor's Score, Driving Recorder Data, Wearable Wireless Sensor Scores According to Leukoaraiosis Grade and Brain Atrophy

**Figure 3:**
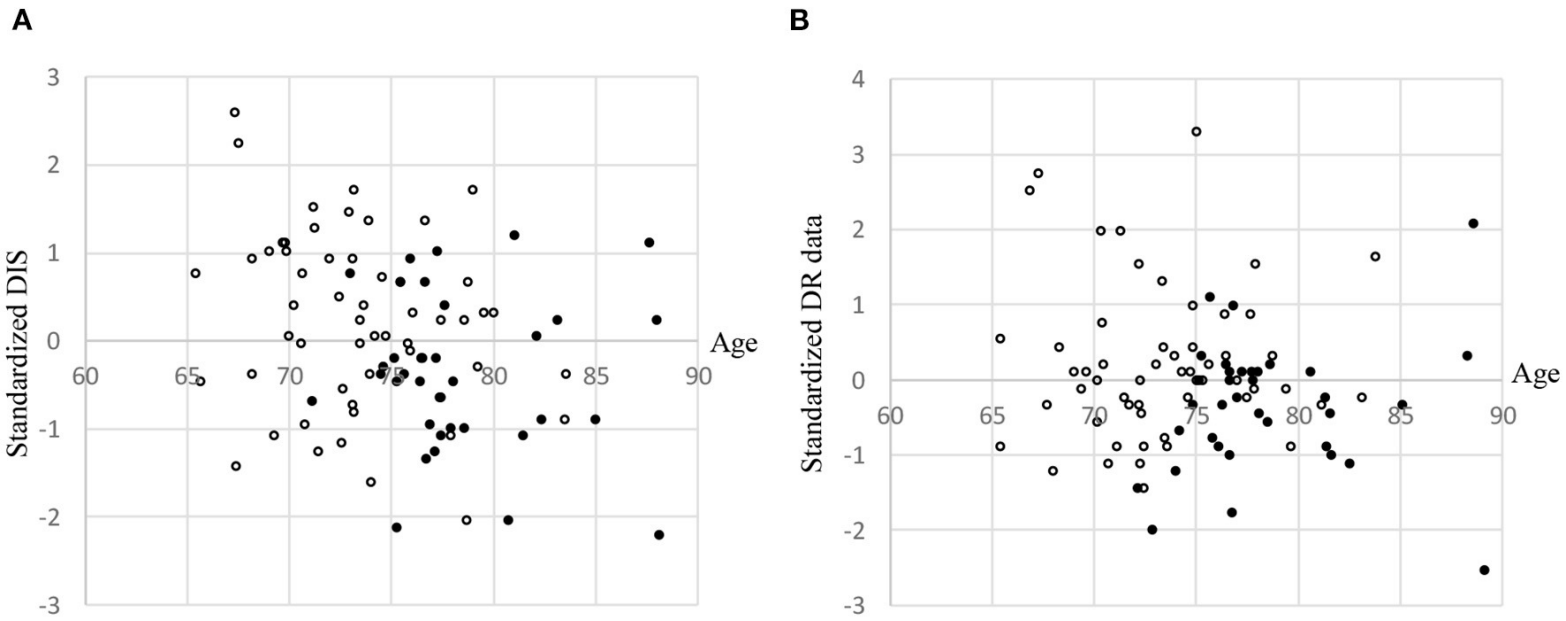
Scatter plots stratified according to leukoaraiosis (LA) grade and brain atrophy (BA). **(A)** Driving Instructor's Score (DIS). **(B)** Driving recorder (DR) data. •: LA ≥ 2 and BA ≥ 0.2176. ∘: All others. DIS and DR data were standardized. In order to avoid overlapping dots, small random values (*M* = 0, *SD* = 0.5) were added to each participant's age.

**Figure 4:**
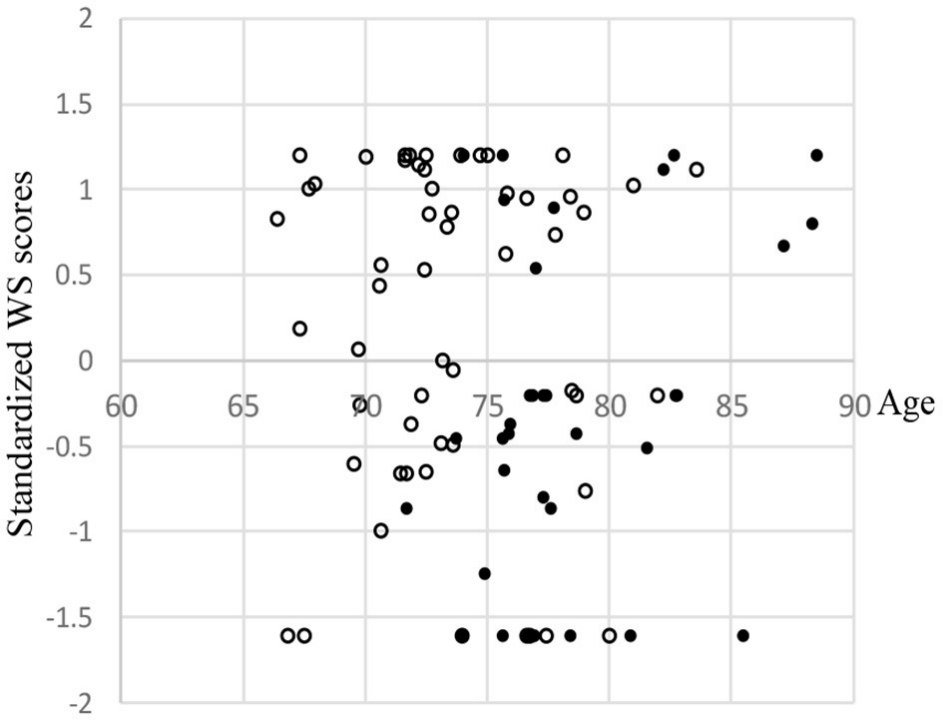
Scatter plots of wearable wireless sensor (WS) scores stratified according to leukoaraiosis (LA) grade and brain atrophy (BA). •: LA ≥ 2 and BA ≥ 0.2176. ∘: All others. WS scores were standardized. In order to avoid overlapping dots, small random values (*M* = 0, *SD* = 0.5) were added to each participant's age.

The correlation coefficients between age and DIS, and those between age and DR data were −0.28 (*p* < 0.01) and −0.09, respectively (Figure 3). DIS and DR data were used for multivariate linear regression analysis. WS scores were binarized as the threshold of the averaged value (Figure 4). The dichotomy was used for logistic regression analysis.

### Regression Analysis Results With Driving Instructor's Score and Driving Recorder Data, and Logistic Regression Analysis Results With Wearable Wireless Sensor Scores

The regression analysis results on DIS and DR data, and the logistic analysis results on WS scores are summarized in Tables 2–4, respectively. These results demonstrate that participants with higher grades of aging brains coupling LA and BA consistently showed significantly larger degradations of DSPs regardless of actual ages (Model 8 in Tables 2–4). The inclusion of both LA and BA revealed that DIS, DR data, and WS scores were significant negative predictors of driving performance when LAs ≥ 2 (Model 7 in Tables 2–4). On the other hand, DIS and DR data were slightly significant (*p* < 0.1) and WS scores not significant when BAs ≥ 0.217. In cases of single LAs and BAs, DIS were not significantly degraded although DR data and WS scores for single LAs were significantly degraded (Models 1 and 6 in Tables 2–4). Additionally, the volumes of frontal, temporal, parietal, and occipital lobes did not individually serve as significant predictors of DSP degradation (Models 2–5 in Tables 2–4).

**Table 2:** Regression analysis results with driving instructor's scores (DIS).

**Predictive variables**	**Objective variable** **=** **driving instructor's score**^g^															
	**Model 1**		**Model 2**		**Model 3**		**Model 4**		**Model 5**		**Model 6**		**Model 7**		**Model 8**	
	**beta**	**s.e**.	**beta**	**s.e**.	**beta**	**s.e**.	**beta**	**s.e**.	**beta**	**s.e**.	**beta**	**s.e**.	**beta**	**s.e**.	**beta**	**s.e**.
**Gender** = Male	0.14	0.10	0.14	0.11	0.17	0.10	0.16	0.10	0.15	0.10	0.18^†^	0.10	0.17^†^	0.10	0.16	0.10
**Age** ^a^	−0.24^*^	0.10	−0.28^*^	0.11	−0.23^*^	0.11	−0.24^*^	0.11	−0.27^**^	0.10	−0.19	0.12	−0.14	0.12	−0.15	0.12
**Brain conditions**																
1) LA^b^ ≥ 2	−0.20	0.10											−0.21^*^	0.10		
2) Frontal_Vol/ICV^c^			−0.03	0.11												
3) Temporal_Vol/ICV					0.13	0.11										
4) Parietal_Vol/ICV							0.09	0.11								
5) Occipital_Vol/ICV									0.10	0.10						
6) BA^d^ = 1 – (TBV^e^/ICV)											−0.2	0.12	−0.17^†^	0.12		
7) LA ≥ 2 and BA ≥ 0.217^f^															−0.24^*^	0.12
**Model statistics**																
*R* ^2^		0.14		0.10		0.12		0.11		0.11		0.12		0.16		0.14
Adjusted *R*^2^		0.11		0.07		0.08		0.08		0.08		0.09		0.12		0.11
*n*		90		90		90		90		90		90		90		90

† *p < 0.1*.

* *p < 0.05*.

** *p < 0.01*.

a *Age is dealt with as a continuous variable*.

b *Leukoaraiosis. Theoretical range = 0–5*.

c *Intracranial Volume*.

d *Brain atrophy*.

e *Total Brain Volume*.

f *The median value was 0.217*.

g *Summation of scores evaluated by an instructor at four points in the driving course. Theoretical range was 0–100. M = 64.2 and SD = 11.4*. *s.e., standard error*.

**Table 3:** Regression analysis results with driving recorders (DR).

**Predictive variables**	**Objective variable** **=** **driving recorder (DR) data**^g^							
	**Model 1**	**Model 2**	**Model 3**	**Model 4**	**Model 5**	**Model 6**	**Model 7**	**Model 8**
	**beta ± s.e**.	**beta ± s.e**.	**beta ± s.e**.	**beta ± s.e**.	**beta ± s.e**.	**beta ± s.e**.	**beta ± s.e**.	**beta ± s.e**.
**Gender** = Male	0.00 ± 0.10	0.01 ± 0.11	−0.01 ± 0.11	0.02 ± 0.11	0.01 ± 0.11	0.05 ± 0.11	0.05 ± 0.10	0.04 ± 0.10
**Age** ^a^	−0.05 ± 0.11	−0.09 ± 0.11	−0.13 ± 0.12	−0.06 ± 0.11	−0.08 ± 0.11	0.00 ± 0.13	0.06 ± 0.13	0.08 ± 0.12
**Brain condition**								
1) LA^b^ ≥ 2	−0.27 ± 0.11^*^						−0.29 ± 0.11^**^	
2) Frontal_Vol/ICV^c^		0.00 ± 0.12						
3) Temporal_Vol/ICV			−0.05 ± 0.12					
4) Parietal_Vol/ICV				0.08 ± 0.12				
5) Occipital_Vol/ICV					−0.01 ± 0.11			
6) BA^d^ = 1 – (TBV^e^/ICV)						−0.21 ± 0.13^†^	−0.24 ± 0.12^†^	
7) LA ≥ 2 and BA ≥ 0.217^f^								−0.33 ± 0.12^**^
**Model statistics**								
*R* ^2^	0.09	0.01	0.03	0.02	0.01	0.07	0.16	0.09
Adjusted *R*^2^	0.04	−0.04	−0.01	−0.03	−0.04	0.03	0.10	0.04
*n*	90	90	90	90	90	90	90	90

† *p < 0.1*.

* *p < 0.05*.

** *p < 0.01*.

a *Age is dealt with as a continuous variable*.

b *Leukoaraiosis. Theoretical range = 0–5*.

c *Intracranial Volume*.

d *Brain atrophy*.

e *Total Brain Volume*.

f *The median value was 0.217*.

g *Summation of the duration (in seconds) of the direction indication during turns at three points in the driving course. M = 17.2 s and SD = 10.7 s. Cronbach's alpha = 0.66*. *s.e., standard error*.

**Table 4:** Regression analysis results with wearable wireless sensor (WS).

**Predictive variables**	**Objective variable** **=** **wearable wireless sensor score**^g^															
	**Model 1**		**Model 2**		**Model 3**		**Model 4**		**Model 5**		**Model 6**		**Model 7**		**Model 8**	
	**beta**	**s.e**.	**beta**	**s.e**.	**beta**	**s.e**.	**beta**	**s.e**.	**beta**	**s.e**.	**beta**	**s.e**.	**beta**	**s.e**.	**beta**	**s.e**.
**Gender** = Male	0.04	0.11	0.07	0.113	0.07	0.11	0.04	0.109	0.05	0.11	0.10	0.11	0.10	0.11	0.07	0.10
**Age** ^a^	−0.01	0.11	−0.02	0.114	0.00	0.11	−0.07	0.113	−0.05	0.108	0.10	0.13	0.15	0.13	0.12	0.12
**Brain condition**																
1) LA^b^ ≥ 2	−0.22^*^	0.11											−0.23^*^	0.10		
2) Frontal_Vol/ICV^c^			0.08	0.118												
3)Temporal_Vol/ICV					0.12	0.12										
4) Parietal_Vol/ICV							−0.06	0.114								
5) Occipital_Vol/ICV									0.03	0.108						
6) BA^d^= 1– (TBV^e^/ICV)											−0.3^*^	0.13	−0.28^*^	0.12		
7) LA ≥ 2 and BA ≥ 0.217^f^															0.33^**^	0.12
**Model statistics**																
*R* ^2^		0.05		0.01		0.02		0.01		0.01		0.05		0.10		0.08
Adjusted *R*^2^		0.02		−0.02		−0.02		−0.03		−0.03		0.02		0.06		0.05
*n*		90		90		90		90		90		90		90		90

* *p < 0.05*.

** *p < 0.01*.

a *Age is dealt with as a continuous variable*.

b *Leukoaraiosis. Theoretical range = 0–5*.

c *Intracranial Volume*.

d *Cerebrospiral Fluid*.

e *Total Brain Volume*.

f *The median value was 0.217*.

g *Summation of two scores on the sufficiency of scanning in the right direction with respect to angle and duration at Point 7 in the driving course. Theoretical range = 0–20. M = 11.46 and SD = 7.12. Cronbach's alpha = 0.97*.

### Relationships Between Brain Conditions and Degradation of Driving Safety Performances in Driving Instructor's Scores

**Figure 5:**
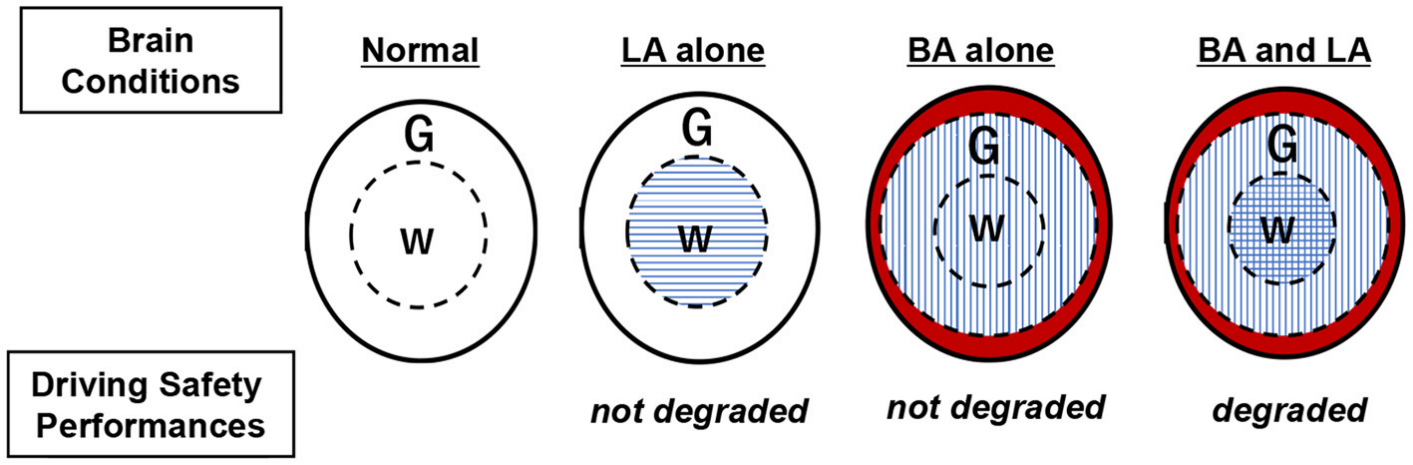
Relationships between brain conditions and degradation of driving safety performances. BA, brain atrophy; LA, leukoaraiosis; G, gray matter; W, white matter. Horizontal line zone shows ischemic change in G due to LA. Vertical line zone shows G and W affected by BA. Red-colored zone indicates brain shrinkage due to BA.

LA is involved in the white matter while BA in the white and gray matter. Brain conditions are defined as normal, LA alone, BA alone, and both of BA and LA. The degradation of DSPs with DIS was observed only in the brain condition of both BA and LA (Figure 5).

## Discussion

In Japan, the rapid increase of traffic crashes involving elderly drivers (Nishida, 2015) is a serious problem in spite of the gradual decrease in numbers of total traffic crashes over all generations, and drivers ≥ 75 years of age, must undergo cognitive function tests when applying for driving license renewals since 2008 (The Driver's License Skill Test for the Elderly Available at National Police Agency, 2016). There are different characteristics of traffic crashes by young and elderly drivers (Cooper, 1990; Abou-Raya and ElMeguid, 2009). Traffic crashes by young drivers are mainly due to excessive speeding while elderly drivers often cause crashes at cross-roads regardless of their speed. Recently, the number of tragic crashes peculiar to elderly drivers and not seen in young drivers is increasing: wrong-entry into highways and incorrect operation of accelerator and brake pedals (Cooper, 1990). Conventional countermeasures for traffic crashes include: heightened awareness of traffic rules, punitive approaches to drunk drivers, improved road infrastructure, increased use of seatbelts, and enhanced vehicle safety (Kim et al., 2015; Nishida, 2015). In addition, measures are required to deal with the peculiar driving attributes of elderly drivers, that is, the decline in executive function that quickly responds to dangerous situations (Cooper, 1990; Abou-Raya and ElMeguid, 2009). While cognitive tests help to improve the knowledge of road rules, there are no medical tests, other than routine eye examinations, that are combined with physical (motor function) tests such as the assessment of the 21 driving skills (mandatory in Japan) in retraining courses for elderly drivers (The Driver's License Skill Test Implementation Standard Available at National Police Agency, 2016). In this study we assume that the “brain” plays a major and essential role in vehicle driving. It is known that individual differences in physical and mental dysfunction increase with aging. Second, individual differences in DSP degradation among older drivers can be attributed to differences in aged brains. Therefore, with the aim of clarifying the relationship between the brain condition of the elderly and DSP, we conducted a driving experiment in an actual vehicle based on an actual Japanese driver's license renewal training test. At the time of unprecedented aging in Japanese society, we propose a new kind of measure using MRI analysis based on a combination of measurable brain attributes, such as BA, LA, and DSP assessments.

A previous study showed a significant correlation between DIS, DR data, and WS scores on a closed- circuit course using the same measurable conditions as in the present study (Renge et al., 2020) indicating that DR and WS may play a complementary role in driving instructors' evaluations. This study revealed that aging brain features such as BA and LA correlate to the degradation of DSP evaluated with DIS, DR data, and WS scores. This result provides hope that aging brain measures using MRI may prevent traffic crashes. However, there is no report directly describing the association of DSP degradation with traffic crashes. From an analysis of the Japanese traffic accident database, drivers who frequently commit traffic violations tend to be more prone to cause traffic crashes (Elbejjani et al., 2019). Traffic violations are naturally considered to occur if drivers do not follow the Road Traffic Act checked items of the DIS evaluation. Although driving with an instructor on a closed driving course is rather different from free driving on ordinary roads, aging brains featuring BA and LA may have an additional impact on the probability of traffic crashes. In order to prove this hypothesis, a large-scale cohort study will be required that examines the direct relationship between traffic crashes and aging brains analyzed using MRI. In this context, MRI may be the most adequate medical tool since it is non-invasive and can be used repeatedly on healthy people. As a result, MRI can provide longitudinal BA and LA data. On the other hand, the present study focused on elderly individuals with BA and LA, two attributes characteristic of aging brains rarely seen in younger people (Guo et al., 2017). Therefore, a more comprehensive comparison using longitudinal studies requires experiments involving wider age ranges of drivers possessing various driving experiences and aging brains.

The present study indicated that no single cerebral lobe showed significant responses related to DSP degradation, suggesting the involvement of whole brains rather than localized brain regions. This suggestion supports our previous result that LA (histological damage in white matter functioning as a neuronal network) is a negative factor regarding drivers' performances regardless of cerebral lobe location under the multitask load of both driving and performing mathematical calculations (Nakano et al., 2014). DSPs may be derived from the functional integration of the whole brain rather than by localized region functions involving LA locations (Wu, 2015; Zhu et al., 2020). Assuming that the involvement of the whole brain consisting of gray and white matter is correct, conceptual diagrams are presented in Figure 5 showing the relationship between brain conditions and the degradation of DSP. In the present study, without a distracting task, LA alone and BA alone led to no significant degradation of DSPs (Models 1 and 6 in Table 2). However, when BA and LA are coupled, they were significantly correlated to the degradation of DSPs (Model 8 in Table 2). LA involves white matter not gray matter. BA represents the changes over the whole brain including both gray and white matter. Therefore, BA possibly produced a degradation effect similar to the calculation-load on DSPs of elderly drivers with LA alone in the previous study. It is now assumed that BA and LA, as different quotients of aging brains, may interact with each other in terms of DSP degradation.

The present study has demonstrated that MRI examinations combined with quantitative perspectives of brain aging contribute to identifying potentially dangerous elderly drivers among healthy ones at the time of driving license renewals. The typical examples of brain healthcare for suppressing BA and LA are lifestyle improvements such as not smoking, drinking responsibly and weight control, in addition to medical treatments such as taking antihypertensive or diabetic drugs. Brain healthcare may be a useful countermeasure toward maintaining DSPs for elderly drivers through the control of BA and LA. The present study may be a small initial step, but has large implications for correlating brain healthcare and risk management for traffic crashes.

### Limitation and Future Works

Toyota Central Research and Keio University teams investigated the relationship between brain and driving performances with 39 and 32 participants, respectively (Sakai et al., 2012; Yamamoto et al., 2020b). The present study enrolled 90 participants, which may be not regarded small but relatively large for the analyses of driving performances using actual motor vehicles connected with MRI data. It goes without saying that larger samples are statistically validated.

DIS was obtained by a publicly qualified driving instructor. As human has less objectivity than machine, DR data and WS scores in addition to DIS were compensatorily used to minimize the less objectivity. Three types of data analyses similarly showed that aging brain degrades DSPs of the elderly. The objectivity may be limitedly validated with DR data and WS scores.

The applicability of the present study may be limited due to very low R (Statistical Handbook of Japan, 2019) values indicating a poor fit in the model with aging brain attributes and DSP. For improved results, it may be useful to combine a specific subdivided cortical region for VBM and a specific DIS category composed of six categories: visual search behavior, speeding, signaling, vehicle stability, positioning, and steering. The combination of single category scores with a specific cortical volume should be investigated for a clearer correlation of drivers' performances. For example, positioning scores may be specifically paired with the parietal areas involved in spatial cognition. In addition, the present study examined DSPs in a closed-circuit course under instructor's inspection, which does not necessarily reflect actual and free driving performances on ordinary roads. The present results should be validated by data analyses of usual driving performances on private cars and ordinary roads.

The present study has focused on elderly drivers because BA and LA (signs of aging brains), are frequently detected in elderly persons and the number of traffic crashes by elderly drivers in Japan has dramatically increased. In fact, we already confirmed that LA is significantly correlated to traffic crashes and low steering skills at intersections (Park et al., 2013; Nakano et al., 2014). Therefore, we developed the idea of assessing BA and LA of the elderly as the first step in understanding the correlation of driving performance with age. Since non-elderly persons infrequently have DA and LA, all generation should be investigated for validation. We are planning a large scaled-experiment with non-elderly persons in near future.

The present study was examined with a driving instructor on the close-circuit course. There should be a big difference in free driving performance on ordinary roads as compared with closed-circuit course under the supervision of a driving instructor. This challenge is a hard barrier to overcome for validation.

All participants were interviewed about traffic crashes over the last 3 years. Two participants with higher grade of aging brain had collision crashes at crossroads and one participant with lower grade had a rear-end collision (data not shown). Larger samples are needed to elucidate the relationship between traffic crashes and aging brain.

The BA and LA are evaluated by structural data of brain. Functional MRI studies elucidate the relationship between LAs and malfunction of neuronal networks because the damaged vessels in LA induce an insufficient blood supply in the white matter including neuronal fibers. BA coexists with dementia and is considered as a cause of cognitive impairment. In order to accurately understand the involvement of brain, it is necessary to collect data on both the structure and the function of brain. We are now planning an experimental approach by using functional MRI and near-infrared spectroscopy (NIRS) to obtain functional data.

## Conclusion

Over all, we showed that aging brains, brain atrophy and leukoaraiosis, evaluated by MRI examination degrade driving safety performances (DSPs) of elderly drivers without dementia (63 men and 27 women, mean age 75.31 ± 4.795 years) using driving instructors' scores, driving recorders' data, and wearable wireless sensors' data on actual vehicles running through a closed-circuit course. MRI examinations have the potential to help identify elderly drivers with dangerous driving behaviors. Brain healthcare, lifestyle improvements and medical treatments to suppress brain atrophy and leukoaraiosis, may contribute to preventing the DPS degradation of elderly drivers with aging brains.

## Data Availability Statement

The data that support the findings of this study are available on the request to the corresponding author.

## Ethics Statement

The studies involving human participants were reviewed and approved by Kochi University of Technology (application number: C4-3). The patients/participants provided their written informed consent to participate in this study. Written informed consent was obtained from the individual for the publication of any potentially identifiable images.

## Author Contributions

KP wrote the main manuscript text, recruited all participants, and collected the questionnaires. KR conducted the research design and collected data. YN conducted all of the analyses and prepared the figures and tables. FY is responsible for processing the MRI images. MT and YK provided insight on statistical analyses and logical configurations. All authors contributed to the article and approved the submitted version.

## Funding

The present study was conducted primarily under the auspices of a research fund of The General Insurance Association of Japan and partially supported by JSPS KAKENHI Grant Number 26285147 and 20H00267.

## Conflict of Interest

The authors declare that the research was conducted in the absence of any commercial or financial relationships that could be construed as a potential conflict of interest.

## Publisher's Note

All claims expressed in this article are solely those of the authors and do not necessarily represent those of their affiliated organizations, or those of the publisher, the editors and the reviewers. Any product that may be evaluated in this article, or claim that may be made by its manufacturer, is not guaranteed or endorsed by the publisher.
